# Early Responses to Severe Drought Stress in the *Arabidopsis thaliana* Cell Suspension Culture Proteome

**DOI:** 10.3390/proteomes6040038

**Published:** 2018-10-02

**Authors:** May Alqurashi, Marco Chiapello, Chantal Bianchet, Francesco Paolocci, Kathryn S. Lilley, Christoph Gehring

**Affiliations:** 1 Department of Biochemistry, Cambridge Centre for Proteomics, Cambridge System Biology Centre, University of Cambridge, Tennis Court Road, Cambridge CB2 1QR, UK; chiapello.m@gmail.com (M.C.); ksl23@cam.ac.uk (K.S.L.); 2 Biological and Environmental Sciences and Engineering Division, King Abdullah University of Science and Technology, Thuwal 23955-6900, Saudi Arabia; c.a.gehring@molecular-signals.com; 3 Department of Chemistry, Biology & Biotechnology, Borgo XX giugno 74, 06121 Perugia, Italy; chantal.bianchet@studenti.unipg.it; 4 CNR, Institute of Biosciences and Bioresources, Perugia Division, Via Madonna Alta, 130 06128 Perugia, Italy; francesco.paolocci@ibbr.cnr.it

**Keywords:** abiotic stress, drought, ribosome, oxidative phosphorylation, endosomal sorting complex

## Abstract

Abiotic stresses are considered the most deleterious factor affecting growth and development of plants worldwide. Such stresses are largely unavoidable and trigger adaptive responses affecting different cellular processes and target different compartments. Shotgun proteomic and mass spectrometry-based approaches offer an opportunity to elucidate the response of the proteome to abiotic stresses. In this study, the severe drought or water-deficit response in *Arabidopsis thaliana* was mimicked by treating cell suspension callus with 40% polyethylene glycol for 10 and 30 min. Resulting data demonstrated that 310 proteins were differentially expressed in response to this treatment with a strict ±2.0-fold change. Over-representation was observed in the gene ontology categories of ‘ribosome’ and its related functions as well as ‘oxidative phosphorylation’, indicating both structural and functional drought responses at the cellular level. Proteins in the category ‘endocytosis’ also show significant enrichment and this is consistent with increased active transport and recycling of membrane proteins in response to abiotic stress. This is supported by the particularly pronounced enrichment in proteins of the endosomal sorting complexes that are required for membrane remodelling. Taken together, the findings point to rapid and complex physiological and structural changes essential for survival in response to sudden severe drought stress.

## 1. Introduction

Drought or water deficit is a major abiotic stress affecting the growth and development of plants. It is the most significant negative factor that affects crop production [1]. Given the increasingly scarce water resources in many areas in the world and the cost of seawater desalination [2,3], understanding drought stress and its effect on plants is of critical economic importance.

The sessile nature of plants necessitated the evolution of specific adaptive responses to both short- and long-term drought stress, which shape different cellular processes and target different cellular machineries. The downstream effect of drought in plants includes reduced cell division and expansion rate, leaf size and root proliferation, impaired enzyme activities, loss of turgor, and decreased energy supply [4,5,6].

In addition, drought also triggers changes in plant hormone homeostasis by decreasing growth promoters such as auxin (IAA) while conversely, growth inhibiting hormones—in particular abscisic acid (ABA)—increase in concentration [7]. The increase in ABA induces stomatal closure to minimise water loss via transpiration [8,9] and controls downstream physiological and developmental processes required for adaptation to stress [10,11,12,13].

Different approaches have been used to induce drought stress in plants including withholding water from soil, air-drying, or the use of chemicals such as mannitol, sorbitol, and polyethylene glycol (PEG). Unlike mannitol and sorbitol which are considered small sugar alcohols, PEG is a high molecular weight solute that cannot enter the apoplastic space of the cell and therefore does not cause lasting and irreversible damage of the cell walls [14]. Polyethylene glycol is known to trigger the severest water potential effect on the cell compared to other chemicals and can mimic drought stress induced by air-drying [14,15,16,17]. The cell suspension culture system was chosen instead of leaves or roots of *Arabidopsis thaliana* for the following reasons. The cell suspension culture offers a unique opportunity to treat a uniform population of cells directly and simultaneously (especially with a liquid-form treatment), rather than multicellular tissue more indirectly, at times differing according to their location in a tissue and/or organ. In addition, because of the comparatively large amount of biological material required for a total proteome study, it is best undertaken with suspension culture cells. Although the findings based on such an experimental set-up reflect cellular changes in this particular model system, they may not necessarily apply to other tissues and/or in whole plants in general. Nevertheless, the system offers significant advantages and has served as an apt and broadly used experimental plant system for e.g., transcriptomic and proteomic studies plant [18].

The complexity of the plant response to environmental stress needs to be investigated not just at the physiological and molecular level, but also at the systems level where global changes during different biological responses can afford insights into their complexity. To the best of our knowledge, no one reported the proteomics profile of early drought responses (i.e., less than one-hour treatment) in semi-differentiated cells such as callus. Therefore, this study examined the effect of drought on the experimental model system of *Arabidopsis thaliana* cell suspension culture using quantitative mass spectrometry-based proteomics.

## 2. Materials and Methods

### 2.1. Plant Material and Growth Conditions

*Arabidopsis thaliana* ecotype Columbia-0 (Col-0) cell suspension culture (root-derived) was kindly obtained from Dr Xiaolan Yu from Professor Paul Dupree’s group, University of Cambridge [19]. The cell suspension culture was grown in Climo Shaker ISF1-X (Kuhner Shaker, Basel, Switzerland) in 250 mL Erlenmeyer flasks containing 100 mL of Gamborg’s B5 medium with vitamins (Sigma-Aldrich, St. Louis, MO, USA) [20] supplemented with 3% (*w/v*) sucrose, 0.05 µg·mL^−1^ (*v/v*) kinetin, and 1 mg mL^−1^ 2,4-dichlorophenoxyacetic acid and pH of 5.7. Cells were grown under light intensity of 100 µmol m^−2^ s^−1^, light/dark cycle of 16/8 h at 21 °C to 25 °C and orbital agitation at 110 revolutions-per-minute. Subculturing (30% of the culture transferred into fresh 250 mL flask with 100 mL medium) occurred every seven days.

### 2.2. Stress Treatment and Protein Extraction

At seven days post-subculturing, three biological replicate flasks were treated with 40% PEG-6000 (Sigma-Aldrich) in media and cells of both the mock treatment (equal volume of milliQ water) and PEG treatment were collected at 0, 10, and 30 min post-treatment. The media containing-PEG were drained off using Stericup^®^ filter unit (Millipore, Billerica, MA, USA), and the cells were immediately snap frozen in liquid nitrogen and stored at −80 °C until use. Approximately 3 g of cells were homogenised using T25 digital Ultra TURRAX (IKA, Staufen, Germany) and proteins were precipitated in 10% trichloroacetic acid in acetone, vortexed, and incubated overnight. Precipitated proteins were pelleted, washed, and re-suspended in urea lysis buffer (7 M urea, 2 M thiourea, and phosphate inhibitor cocktail 2 (product number P5726); Sigma-Aldrich).

### 2.3. Peptide Labelling with Tandem Mass Tag

Approximately 100 µg of total soluble protein extract samples were adjusted in volume to 100 µL with 50 mM 4-(2-hydroxyethyl)-1-piperazineethanesulfonic acid (HEPES; pH 8.0). Reduction with 10 mM dithiothreitol in HEPES (pH 8.0) was applied and incubation took place for one hour at 37 °C, followed by alkylation with 25 mM iodoacetamide in HEPES (pH 8.0), incubation for two hours in dark and precipitation with 10 volumes of cold acetone overnight −20 °C. The samples were then centrifuged at 16,000 *g*, supernatant was decanted, and pallets were re-suspended in 100 mM HEPES (pH 8.5) and sonicated. After that, samples were subjected to digestion with trypsin in HEPES (pH 8.5) 1:40 ratio over-night at 37 °C, label with isobaric tandem mass tag (TMT™) ten-plex (Thermo Fisher Scientific, Waltham, MA, USA) for two hours at room temperature, quench with 8 µL of 5% hydroxylamine in 100 mM HEPES (pH 8.5) for one hour, and then further quenched with 100 µL water overnight at 4 °C. Each biological replicate was labelled separately then pooled together for further analysis. In a final step, the pooled TMT labelled peptides were desalted using Sep-Pak Vac tC18 100 mg cartridge (Waters, Milford, MA, USA). The desalted sample was then dried with a Speed Vac concentrator.

### 2.4. High pH Reversed Phase Chromatography Fractionation

The TMT sample was subjected to off-line chromatography fractionation step with mobile phase A composed of 20 mM ammonium formate, pH 10.0 and mobile phase B composed of 20 mM ammonium formate, pH 10.0 in 80% (*v/v*) acetonitrile. As a standard, 42 pmol/µL digested α-casein was diluted 1:10 in mobile phase A and run prior to the samples. 

Dried peptides were re-suspended in mobile phase A and injected on to an Acquity ultra-performance liquid chromatography (UPLC) BEH C18 column (Waters) (1.7 μm particle size, 2.1 mm inner diameter × 150 mm length) in a Waters Acquity UPLC off-line chromatography system (Waters). The chromatography system was primed in 95% mobile phase A and 5% mobile phase B prior to any injection. The flow rate for elution was set to 0.244 mL/min and a constant column temperature of 40 °C. Peptides were eluted using a linear gradient from 5% B to 75% B over 50 min (the total LC gradient run time including washing and re-equilibration was 75 min). Peptide elution was monitored using a photodiode array (PDA) detector, which scanned between 210 and 400 nm, and fractions were collected in one-minute intervals using a fraction collector. Peptide fractions were dried to completion in a Speed Vac concentrator and stored at −80 °C until use.

### 2.5. Protein Identification by Mass Spectrometry and Quantification of Differentially Expressed Proteins

Out of the 50 collected fractions for the TMT sample, the 35 peptide-containing fractions were re-suspended in 10 µL of 0.1% (*v/v*) formic acid and pooled in a pair-wise manner (16 fractions in total) in preparation for mass spectrometry analysis.

#### TMT-Labelled Quantification

The re-suspended fractions (5-µL injection of around 0.29 µg/µL per fraction) were run on Dionex Ultimate 3000 RSLC nanoUPLC (Thermo Fisher Scientific) system and a QExactive Orbitrap mass spectrometer (Thermo Fisher Scientific). Separation of peptides was performed by reverse-phase chromatography at a flow rate of 300 nL/min and a Thermo Scientific reverse-phase nano easy-spray column (Thermo Scientific PepMap C18, 2 µm particle size, 100A pore size, 75 µm i.d. × 50 cm length). Peptides were loaded onto a pre-column (Thermo Scientific PepMap 100 C18, 5 µm particle size, 100A pore size, 300 µm i.d. × 5mm length) from the Ultimate 3000 autosampler with 0.1% formic acid for 3 min at a flow rate of 10 µL/min. After this period, the column valve was switched to allow elution of peptides from the pre-column onto the analytical column. Solvent A was water and 0.1% formic acid while solvent B was 80% acetonitrile, 20% water and 0.1% formic acid. The linear gradient employed was 2–40% B in 100 min (120 min total run time).

The LC eluent was sprayed into the mass spectrometer by means of an easy-spray source (Thermo Fisher Scientific). All *m*/*z* values of eluting ions were measured in an Orbitrap mass analyser, set at a resolution of 70,000. Data dependent scans (Top 20) were employed to automatically isolate and generate fragment ions by higher energy collisional dissociation (HCD) in the quadrupole mass analyser and measurement of the resulting fragment ions was performed in the Orbitrap analyser, set at a resolution of 17,500. Peptide ions with charge states of 2+ and above were selected for fragmentation.

All spectra were submitted for protein identification to MASCOT search engine with *Arabidopsis thaliana* TAIR10 database. A precursor mass tolerance of 20 ppm, a fragment ion mass tolerance of 0.1 Da, peptide charge of up to 4+, allowing up to two missed cleavages and instrument option of ESI-Orbitrap-HCD was applied. Fixed modifications included carbamidomethylation of cysteine residues while variable modifications included TMT six-plex of *N*-terminus, serine, threonine, tyrosine, and lysine residues. Identified proteins were evaluated and quantitated using Proteome Discoverer (v1.4) (Thermo Fisher Scientific).

Proteins were considered as identified with a minimum of two unique peptides, a MASCOT ion score ≥26, a high peptide confidence (FDR 1%), and a peptide rank of maximum 1. The analysis was carried out using MSnbase R package (R v3.3.1) [21]. Differential expression analysis was performed using empirical Bayes moderated *t*-test as implemented in the linear models for microarray data (LIMMA) library (v3.28.14) [22] from Bioconductor (v3.3). Missing values were imputed at peptide level using the hybrid method from imputeLCMD R package (v2.0). The method distinguishes rows (peptides) that contain non-random missing data (KNN method) from those lines that contain random missing data (Min Probability method). Normalisation on peptide level was done using variance stabilising normalisation (VSN) package while median normalisation was applied on a protein level across all samples. Proteins were considered significant if the fold change was at least ±2.0-fold change (±1.0 in log2 transformation) and statistical significance of Benjamini-Hochberg adjusted *p*-value of ≤0.05.

### 2.6. Computational Analysis of Functional Enrichment

The gene ontology (GO) and functional categorization analyses of the differentially expressed proteins were performed using AgriGO online tool and search was performed against *Arabidopsis thaliana* TAIR9 database (http://bioinfo.cau.edu.cn/agriGO/analysis.php, May 2016) [23]. Significant GO terms were selected with adjusted *p*-value of ≤0.05 to correct for multiple hypothesis testing.

Pathway enrichment was performed using Kyoto encyclopaedia of genes and genomes (KEGG) orthology based annotation system (KOBAS) online tool (v2.0) (http://kobas.cbi.pku.edu.cn/home.do, June 2016) [24]. Significant terms were selected with an adjusted *p*-value of ≤0.05 to correct for multiple hypothesis testing.

Over-representation of motifs in the promoter region of the significantly changing proteins was done using the Element online tool (v2.0) (http://element.mocklerlab.org/, June 2016) [25] and searched against the *Arabidopsis thaliana* TAIR10 database.

The online tool Genevestigator (https://genevestigator.com/gv/doc/intro_plant.jsp, June 2016) [26] was utilized to study gene co-expression in experimental databases to identify which conditions affect the gene expression and find similarities of expression between genes and/or between regulating conditions. Network representations were generated using Cytoscape^®^ v3.6.1 [27] while ScanProsite (https://prosite.expasy.org/scanprosite/, May 2018) was used to detect PROSITE signature motif matches [28]. Protein Basic Local Alignment Search Tool (BLAST; https://blast.ncbi.nlm.nih.gov/Blast.cgi, May 2018) was also used to investigate homology of proteins [29]. The Conserved Domain Database (CDD; https://www.ncbi.nlm.nih.gov/cdd/, May 2018) was utilized to identify conserved domains present in proteins [30,31].

### 2.7. Cell Viability Test

To test cell viability, 4 µM fluorescein diacetate (Sigma-Aldrich) was used for staining the control and PEG-treated Arabidopsis cells. The staining solution was added to control cell suspensions (mock treatment), or after 0.5 h treatment with 40% PEG and treated cell suspensions 24 h post-treatment. The samples were incubated for 5 min at room temperature before observation with a fluorescent microscope using a 450 nm excitation filter and a 535 nm emission filter. The total number of Arabidopsis cells was visually counted using bright-field microscopy, while the fluorescent cells in the same field of view were counted using a fluorescence microscope. Three independent experiments were performed and for each experiment, typically >1000 cells were counted per sample. 

## 3. Results

### 3.1. Cell Viability Test

**Figure 1:**
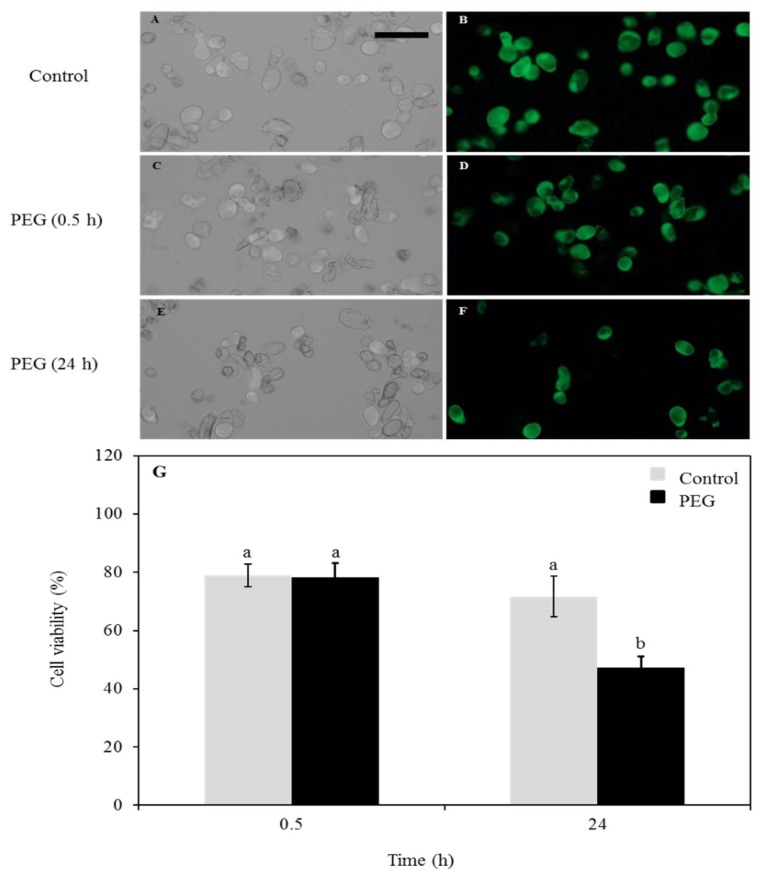
*Arabidopsis thaliana* cell viability assessed with FDA after treatment with 40% PEG. (**A**) Control (mock-treatment) cell count under bright-field microscope. The bar indicates 100 µm. (**B**) Control (mock-treatment) cells under the fluorescent microscope. (**C**) Cells after 0.5 h treatment under bright-field microscope. (**D**) Cells after 0.5 h treatment under fluorescent microscope. (**E**) Cells 24 h post-treatment under bright-field microscope. (**F**) Cells 24 h post-treatment under fluorescent microscope. (**G**) Diagram showing a comparison between control and treated cells after 0.5 h treatment and 24 h post-treatment; a, non-significant change (>0.05); b, significant change (<0.05).

Given the severity of the drought-treatment used in this study (40% PEG-6000), we first established whether the cells could recover from such a treatment or if it would cause irreversible damage and subsequent cell death. To this end, cell viability and apoptosis assays were performed using fluorescein diacetate staining followed by examination with the fluorescent microscopy. The control cells noted the normal properties of callus cell (Figure 1A,B). At 30 min post treatment, the cells showed a high rate of survival similarities to control group (Figure 1C,D). This indicated that the cells have retained their physical integrity and have survived during the time of the treatment. To assess the longer-term effect of the stress on the cells, they were transferred back to PEG-free media after the stress and monitored for 24 h (Figure 1E,F). At this time point, an increased number of dead cells was noted. The cell viability was also measured in percentage and compared between the control and PEG-treated cells across the two measured times and high percentage of survival was noted (Figure 1G) and no signs of plasmolysis or cytorrhysis were observed, indicating cell death after the treatment was not necessarily the default pathway.

### 3.2. The Effect of Drought on the Proteomic Profile

To investigate the effect of early response to severe drought stress on the proteomic profile of the cell, Arabidopsis Columbia-0 (Col-0) cell suspension cultures were treated with 40% PEG and samples collected at 10 and 30 min after the treatment. Proteins extracted from three biological replicates were each subjected to digestion, desalt purification, fractionation, and quantification analysis.

**Figure 2:**
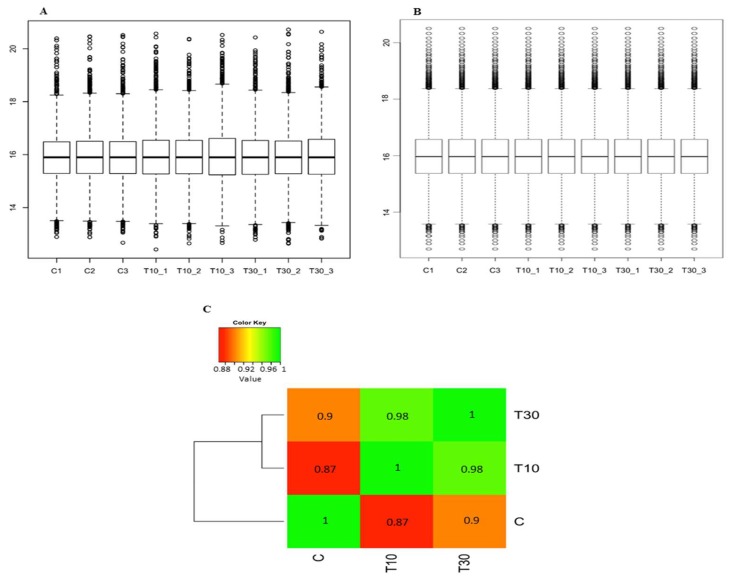
Diagrams showing the distribution of TMT-labelled sample after applying bioinformatics analysis. (**A**) Normalisation on the peptide level using variance stabilising normalisation. (**B**) Normalisation on the protein level using median. (**C**) Heatmap showing normal correlation with distinct distribution between the control and the treated samples. Control, C; 10 min treatment, T10; 30 min treatment; T30.

The raw data files of the TMT-labelled sample identified 6656 quantifiable proteins (Table S1). After applying normalisation on both the peptide level and the protein level (Figure 2A,B), the data showed a normal distribution with distinct distribution between the control and the treated samples (Figure 2C). Moderate *t*-test and Benjamini-Hochberg adjusted *p*-values of ≤0.05 showed 1700 proteins (25.54%) differentially expressed with fold changes ranging from 6.40 to −8.81-fold change (2.68 to −3.14 in log2 transformation) for the 10 min treatment and from 4.72 to −7.06-fold change (2.24 to −2.82 in log2 transformation) for the 30 min treatment (Table S2).

These proteins were also subjected to gene ontology (GO) analysis and ranked in order of the fold change. The range of fold changes have been divided into seven groups namely ±1.2–1.4 (310 proteins), ±1.4–1.6 (674 proteins), ±1.6–1.8 (418 proteins), ±1.8–2.0 (215 proteins), ±2.0–4.0 (295 proteins), ±4.0–6.0 (21 proteins), and ±6.0–9.0 (3 proteins). Terms of ‘biological process’ from the GO analysis that have been enriched in response to the drought stress were observed in high fold change magnitude (±1.8–9.0) and they include ‘ribosome biogenesis’, ‘translation’, ‘response to water deprivation’, ‘response to osmotic stress’, ‘response to cold’, ‘photorespiration’, and ‘mitochondrial ATP synthesis coupled electron transport’. The low fold change magnitude (±1.2–1.8) was enriched in the terms ‘ER to Golgi vesicle-mediated transport’, ‘intracellular transport’, ‘protein import into nucleus’, ‘translational initiation’, and ‘tRNA aminoacylation for protein translation’.

The ‘molecular function’ category of the GO analysis comprises of the lower fold change groups (±1.2–1.8) and are enriched in terms including ‘GTP binding’, ‘ATP binding’, ‘aminoacyl-tRNA ligase activity’, ‘calcium-transporting ATPase activity’, and ‘translation initiation factor activity’. The higher fold change groups (±1.8–9.0) were enriched solely in the category ‘structural constituent of ribosome’.

In order to focus on the most abundant cellular response, a strict ±2.0-fold change (±1.0 in log2 transformation) was applied and yielded 310 proteins (18.23%) differentially expressed after the PEG treatment compared to the control (Table S3).

Gene ontology analysis yielded 10 enriched terms in the ‘biological process’ category including ‘ribosome biogenesis’, ‘translation’, ‘lipid localization’, ‘mitochondrial ATP synthesis coupled electron transport’, ‘photorespiration’, ‘response to cold’, ‘vesicle-mediated transport’, ‘protein folding’, ‘response to osmotic stress’, and ‘response to water deprivation’ with adjusted *p*-values ranging from 2.70 × 10^−18^ to 0.01, respectively. All the terms included proteins that increased in abundance except for ‘ribosome biogenesis’ and ‘translation’ which included proteins that decreased in abundance. The category ‘molecular function’ was enriched in only one term, ‘structural constituent of ribosome’ with an adjusted *p*-value of 1.30 × 10^−34^.

**Figure 3:**
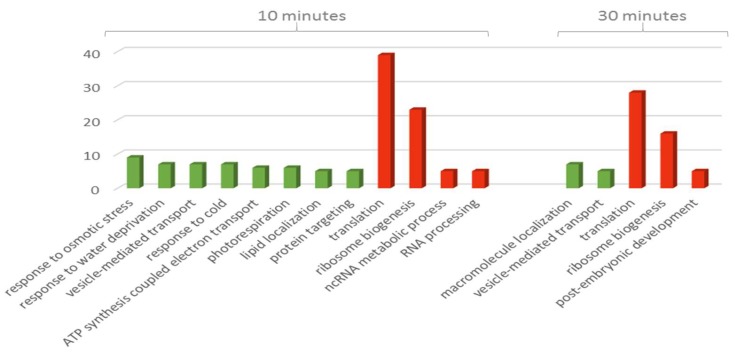
Bar chart showing numbers of proteins ordered according to biological process term at 10 and 30 min treatments of TMT-labelled group. Green bars indicate increased abundance while red bars indicate decreased abundance.

Interestingly, the GO terms enriched in the ‘biological process’ showed a distinct direction of response according to the duration of the treatment (Figure 3). The 10 min time point included all the responses terms (i.e., cold, water deprivation, and osmotic stress) along with ‘mitochondrial ATP synthesis coupled electron transport’, ‘lipid localization’, ‘photorespiration’, ‘ncRNA metabolic process’, and ‘RNA processing’ while the 30 min included ‘macromolecule localization’ and ‘post-embryonic development’. Three terms on the other hand were enriched at both time points, ‘vesicle-mediated transport’, ‘ribosome biogenesis’, and ‘translation’.

Although the ‘molecular function’ category was only enriched in one term, ‘structural constituent of ribosome’, this term was enriched mainly in proteins that decrease in abundance and showed an inverse proportion in the number of proteins against the duration of the treatment (38 proteins after 10 min and 27 proteins after 30 min).

If the 10 proteins with the most significant change in abundance at each treatment time point were considered, at 10 min the proteins increasing in abundance were NADH-ubiquinone oxidoreductase B18 subunit (AT2G02050), prefoldin 6 (AT1G29990), lipid transfer protein 5 (AT3G51600), mitochondrial ATP synthase D chain (AT3G52300), tubulin folding cofactor A (KIESEL; AT2G30410), cytochrome C oxidase biogenesis protein Cmc1-like (AT5G16060), ribosomal protein L31 (AT5G55125), and three unknown proteins (AT5G03660, AT3G05070, and AT4G15790) (Table 1). In contrast, the proteins with the most decreased abundance include eight ribosomal proteins as well as embryo defective 2296 (AT2G18020) and nucleic acid-binding, OB-fold-like protein (AT4G30800) (Table 1). At 30 min, most of the above proteins were included except for lipid transfer protein 3 (AT5G59320), and the defensin-like protein 196 (AT2G43535) that increased in abundance while DEK domain-containing chromatin associated protein (AT5G63550) and ribosomal protein L18e/L15 superfamily protein (AT5G64670) decreased (Table 2).

Pathways enrichment using the KOBAS online tool on the TMT-labelled group proteins yielded three pathways (Table S4). These pathways are ‘Ribosome’ with 48 proteins included and adjusted *p*-value of 2.58 × 10^−24^, ‘oxidative phosphorylation’ containing 18 proteins with adjusted *p*-value of 1.42 × 10^−7^ and ‘endocytosis’ that included 10 proteins and had an adjusted *p*-value of 2.88 × 10^−3^. Interestingly, similar patterns pertaining to the proteins increased or decreased in abundance were observed. The ‘ribosome’ pathway consisted of 40 proteins decreasing in abundance while on the other hand; the ‘oxidative phosphorylation’ and ‘endocytosis’ pathways had all their proteins increased in abundance.

Since transcriptional control is governed by promoters located upstream of the transcribed region of genes, we searched for common motifs within these promoters that may be indicative of the role the respective genes may play in response to external stimuli. Using the element online tool, significantly changing proteins from the TMT-labelled group (Table S3) showed enrichment of motifs in their promoter region. These motifs included ‘SORLIP2’, ‘Dof’, ‘I-BOX’, ‘CAAT/CCAAT-BOX’, ‘WRKY71’, ‘MYB2’, and ‘G-BOX’ among others (Table S5).

Looking at the significantly changing TMT-labelled proteins identified, 17% (53 out of 310 proteins) are annotated as unknown proteins, of which 88.68% (47 proteins) increased in abundance in response to drought stress. Incidentally, three of these unknown proteins, (AT3G05070), (AT4G15790), and (AT5G03660) were also in the ten most regulated proteins (Table 1). A recent study used an integrative annotation pipeline to re-annotate the *Arabidopsis thaliana* reference genome in order to discover novel functions to these previously unknown proteins [32]. Among them were the three unknowns of interest, now annotated as Cwf18 pre-mRNA splicing factor (AT3G05070), uveal autoantigen with coiled-coil/ankyrin (AT4G15790), and transcriptional activator (DUF662; AT5G03660) according to the domains they contain.

**Figure 4:**
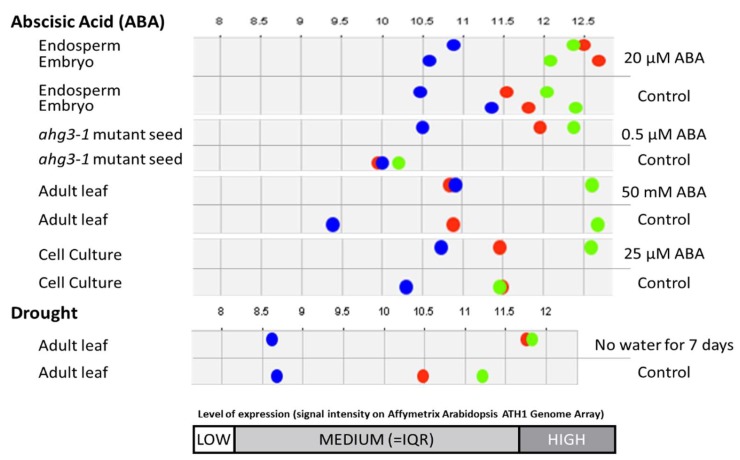
Expression levels the three unknowns of interest showing increased expression level to ABA and drought stress. Protein A (AT3G05070) is represented as a green dot, protein B (AT4G15790) is represented as a blue dot while protein C (AT5G03660) is represented as a red dot.

After utilizing Genevestigator for gene co-expression and condition analysis, these three unknown proteins—hereafter addressed as protein A (AT3G05070), protein B (AT4G15790) and protein C (AT5G03660)—showed an increase in expression in response to ABA and drought stress in different parts of the Arabidopsis plant (Figure 4).

Co-expression analysis performed using Genevestigator indicated several positively co-expressed genes with each of the three genes that encode the unknown proteins of interest in this study (Table 2).

Gene ontology analysis for each of the three unknown genes of interest and their top 10 positively co-expressed proteins showed only protein B to have common terms with its co-expressed genes. The enriched GO terms included ‘translation’ in biological process, ‘structural constituent of ribosome’ in molecular function, and ‘cytosolic ribosome’ in cellular compartment.

Furthermore, BLAST homology searches showed that protein A has similarity to proteins annotated as coiled-coil domain containing protein 12 in different organisms including *Arabidopsis lyrata*, *Brassica napus*, and *Eutrema salsugineum*. Protein B and C show homology to non-muscle myosin heavy chain 3 and non-muscle myosin heavy chain 9, respectively.

Analysis using the Conserved Domain Database showed that protein A contains cwf18 pre-mRNA splicing factor (pfam08315) domain, protein B contains autophagy protein Apg6 (pfam04111) domain, SH3 domain protein (TIGR04211), and chromosome segregation ATPase (COG1196) while protein C contained transcriptional activator (pfam04949) domain.

In addition, the PROSITE online tool enabled the discovery of signature motifs and showed all three unknown proteins to contain protein kinase C (PKC) phosphorylation site and both protein A and B to contain casein kinase II phosphorylation site. Protein B also contained *N*-glycosylation site, leucine zipper pattern, and *N*-myristoylation site while protein C harbours cyclic adenosine monophosphate (cAMP)- and cyclic guanosine monophosphate (cGMP)-dependent protein kinase phosphorylation site.

**Figure 5:**
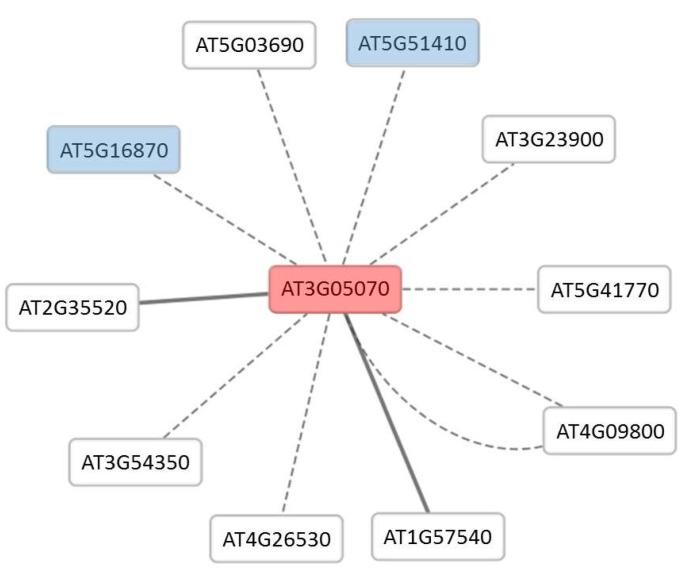
Cytoscape network of protein A interactome. Protein A (AT3G05070) is represented in the centre (red) and two of the interacting proteins (blue) are present in this study. Dotted edges represent predicted interactions and solid edges represent the protein complementary assay interaction.

Further analysis of protein A using Cytoscape generated a network of eight predicted interacting proteins using the Bio-Analytic Resource (BAR) database and two experimentally proven (protein complementation assay) interacting proteins using Agile Protein Interactomes DataServer (APID) database (Figure 5). Two of the predicted proteins are present in this study (Table S2) with (AT5G51410) increase in abundance after 10 min and (ATG16870) decrease in abundance after 10 min. Among the predicted proteins interacting with protein A are two fructose-bisphosphate aldolases (FBPA); FBPA 4 (AT5G03690) and FBPA 5 (AT4G26530). Further GO analysis on this network showed two enriched biological process terms, ‘cellular macromolecule metabolic process’ and ‘gene expression’.

In silico analysis of protein C was performed utilizing an in-house Perl scripts to search for the adenylyl cyclase (AC) catalytic centres motif with the pattern ([RKS].[ED].{9,11}[RK].{1,3}[ED]) in a FASTA file as input. Searching showed protein C and four of its top ten positively co-expressed proteins (Table 2) to have at least one motif in their protein sequence, namely (AT1G29990, AT3G50360, AT4G35570, and AT5G46020).

## 4. Discussion

The nature and type of proteins that are differentially expressed after the severe drought treatment shed light on the early cellular responses. Previous studies have shown that differential expression of genes is the highest at early stages (up to one hour) of drought stress response and will decrease with prolonged stress [33] while ABA starts to accumulate in response to the same stress after two hours [34]. Similar response patterns have been observed in this study at the protein level with number of significantly differentiated proteins reduced between 10 and 30 min. Although many previous studies expressed early drought response from treatments of 1 h to 24 h, it is interesting to observe that such a strong early response can occur just 10 min after the onset of the stress. This may indicate that the drought cellular response is highly specific, efficient, and essential to maintaining cellular homeostasis.

The relationship observed between changes in protein abundance and enriched GO terms related to these proteins is not unexpected, with the signalling components such as ‘response to water deprivation’ and ‘response to osmotic stress’ showing more modest changes in expression and process components such as ‘vesicle-mediated transport’ and ‘ribosome biogenesis’, demonstrating more marked changes in abundance. The over-representation of ribosomal components as well as components with a role in oxidative phosphorylation at an early stage suggest that the ribosomes control, and hence de novo protein synthesis, are essential for a successful defence against the stress. Incidentally, changes in the translational apparatus has been observed previously in *Arabidopsis thaliana* under controlled soil water deficit drought (withholding water for five to seven days) [33].

The translation machinery was observed to have a complex response to drought stress since some ribosomal constituent proteins decreased in abundance thereby contributing to a reduction in protein synthesis, while three translation initiation factors (AT1G66070, AT1G54290, and AT5G37475) and one translation elongation factor (AT3G18760) significantly increased in abundance. This may indicate that translation of some, perhaps highly stable and/or RNA-binding proteins [35] are an essential component of the drought-response.

The pathway analysis further highlights a novel role of ‘endocytosis’ in the early stress response. Members of the small GTP-binding proteins family, namely the Rab/Ypt subfamily have a distinct association with specific intracellular compartments of the endocytic pathways. Rabs are mainly found to be on the cytoplasmic face of organelles and shown to be involved in vesicle formation, transportation, and docking [36]. Some Rab genes were found to have a cellular response to environmental stimuli such as drought stress [37]. In the Arabidopsis genome, there are 57 identified Rab proteins [38] of which 11 were identified in this study with all of them showing decreased abundance in both treatment time points (Table S2). 

Further evidence of the involvement of vesicle trafficking the abiotic stress response, several additional endosomal components are also highlighted by this study. Endosomes are involved in vesicular trafficking from the plasma membrane and the Golgi apparatus towards the lysosome for degradation. Part of this machinery consists of the endosomal sorting complexes required for transport (ESCRT) that contains vacuolar protein sorting-associated (VPS) proteins. They form ESCRTs 0, I, II, and III [39] and are essential for membrane remodelling. ESCRT have essential roles in the biogenesis of the multivesicular bodies (MVB) and the sorting of ubiquitinated membrane proteins in to their intraluminal vesicles (ILVs) for degradation upon the fusion of the MVB and the vacuole/lysosome [40,41].

Almost all of the ESCRT subunits are present in yeast, as well as multicellular organisms, with the noticeable exception of ESCRT-0, which is absent from plants [42]. The Arabidopsis genome has been shown to contain nine TOM1-like (TOL) proteins (orthologues of ESCRT-0 subunits) and some can effectively bind to ubiquitin and enable the internalization and vacuolar sorting of the auxin efflux facilitator [43]. Given the role this pathway has in sorting and downregulating activated cell-surface receptors, changes in the ESCRT component proteins are likely to have direct or indirect effects on physiological and developmental processes.

In this study, four VPS proteins from ESCRT-III (AT2G06530, VPS2.1; AT5G44560, VPS2.2; AT2G19830, VPS32.1 and AT4G29160, VPS32.2), two ESCRT-III associated (AT5G04850, VPS60.2 and AT1G73030, VPS46.2 or CHMP1A) as well as two from ESCRT-I (AT3G53120, VPS37.1 and AT2G36680, VPS37.2) were identified as significantly increased in abundance in response to the drought stress treatment (Table S4). These proteins have been shown to have various roles in development and responses. While CHMP1A knock-down mutants die at the germination stage [44] when in combination with VPS60, they play a regulatory role in the ESCRT-III activity [45]. VPS2 and VPS32 are considered structural components of the ESCRT-III, where VPS32 is part of the ‘core’ sub-complex while VPS2 is part of the ‘coat’ sub-complex [45]. The most divergent component of the ESCRT-I is VPS37 and the yeast homologues are known to interact with VPS20 from ESCRT-III thus linking both complexes [46]. However, such a function in the plant orthologs has still to be determined experimentally. Furthermore, a study has indicated involvement of ESCRT-I in plant immunity where mutant *vps37.1* plants showed enhanced growth of *Pseudomonas syringae* pv. tomato and that bacterial flagellin-induced stomatal closure was significantly impaired [47].

Moreover, a study attempted to explain the important role of ESCRT within in a new concept termed “the crowded place” [48]. The authors describe how the typical Arabidopsis cell surface area is ~600 µm^2^ with 610 different receptor-like kinases, which make the ratio of the receptor-like kinases per surface area approximately one receptor per µm^2^. Therefore, under increased signalling activity, the cell would utilise endosomes as additional inner surface for signalling [48]. A recent review on the non-canonical importance of plant ESCRT machinery has demonstrated that its involvement not only in endosomal sorting of proteins but also in in non-endosomal sorting events such as autophagy, cytokinesis, viral replication and plant-specific processes, ABA signalling, and chloroplast turnover [49]. Given the decreased abundance of Rab proteins and increased abundance of all identified ESCRT proteins in this study, we hypothesise that ESCRT proteins have a role in early drought stress signalling and downstream cellular responses.

Many ABA synthesis and ABA-dependent signalling processes and proteins have been observed to increase in abundance after drought stress including prefoldin proteins [50], annexin proteins that is also associated with cold and water depletion response [51], as well as cold stress-induced proteins (KIN1 and KIN2) [52,53] (Table S3). In addition, several drought-induced proteins such as late embryogenesis abundant (LEA) and early response to dehydration (ERD) were found to be differentially expressed in this study but were not included in the final analysis due to their fold change being below the selected strict threshold of ±2.0-fold change (±1.0 in log2 transformation; Table S2). However, it is conceivable that they are part of a later response (>30 min) as indicated by previous studies [34,54].

The analysis performed on the three unknown proteins in this study, protein A (AT3G05070), B (AT4G15790), and C (AT5G03660) may aid in establishing their function(s) and infer their importance in stress responses. Given that they increase in abundance in response to drought stress as well as show increased transcript levels in response to ABA, this is indicative of a plausible role in drought stress responses.

All three unknowns of interest share similar strong and significant response to the drought stress, as observed in this study in addition to a structural resemblance, as they all contain coiled-coil domain and consist of a relative short protein sequence (144 to 173 amino acids). Protein A functionally clusters with proteins that function as part of the ribosome. For example, it has been predicted to directly interact with a network of proteins such as RNA recognition motif-containing protein, peptidyl-tRNA hydrolase II, 40S ribosomal protein S18 and FBPAs (Figure 4) and has been shown to be co-expressed with ribosomal RNA-processing proteins, the non-catalytic subunit of nuclear DNA-dependent RNA polymerases, and RNA-binding protein and coiled-coil proteins (Table 2). The network therefore implicates protein A in roles in the regulation of mRNA processing from expression to translation, and given the quantitatively strong response (Table 1), we hypothesise that the role is critical to the abiotic stress response. In addition, coiled-coil type proteins have previously been shown to have a role in the regulation of gene expression, notably as transcription factors [55,56,57], and this again is consistent with a role in the rapid transcriptional reprogramming in response to stress. The fact that protein A also contains a cwf18 pre-mRNA splicing factor (pfam08315) domain further supports a role in the reprograming of stress-induced transcriptional activities.

Protein B is co-expressed with genes encoding proteins that are enriched in translational and structural constituents of the ribosome suggesting that they might be part of a response to stabilise translation under the rapid onset of stress. It also contains a leucine zipper that is a motif present in many gene regulatory proteins such as the cAMP response element (CRE) binding proteins [58]. Given the homology shared between this protein and different non-muscle myosin heavy chain 3 in other organisms suggests a myosin-like role. Plant myosins are molecular motor proteins that bind to organelles and interact with actin, which organizes and directs intracellular movement [59]. They can form a network of membrane-anchored receptors that are important in transport and notably cytoplasmic streaming [60] which is almost certainly majorly altered during the rapid onset of dehydration. 

Protein C shows features that may indicate involvement in cell signalling since it contains a transcriptional activator family domain (DUF662) that is involved in salt, drought, cold, ABA, and other stress condition responses [61]. Given that cyclic AMP is one of the most influential second messengers in the cell and has been studied previously to illicit abiotic responses in *Arabidopsis thaliana* [62,63], the fact that protein C contains a cAMP- and cGMP-dependent protein kinase phosphorylation site ([RK]X{2}[ST]) highlights the increasing role of cyclic mononucleotide signalling in a plant’s response to stress.

## 5. Conclusions

Quantitative proteomic approaches using isobaric tagging and mass spectrometry revealed the response of the proteome to stress conditions. This study shows the early profile of drought stress induced in *Arabidopsis thaliana* and 310 of the differentially expressed proteins were analysed with a view to gain new insights into the early responses to severe drought stress. Our results implicate endocytotic processes as a key to early stress signalling and a likely determinant of cell fate under conditions of severe drought stress. This study also highlighted three structurally-annotated unknown proteins and will help direct future studies to their function and roles in stress response.

## Figures and Tables

**Table 1:** Top regulated proteins after 10- and 30-min treatments

	Accession	Annotation	Average 10 min	Average 30 min	Adjusted *p*-Value (10)	Adjusted *p*-Value (30)
**Increased Abundance**	AT3G52300	ATP synthase D chain, mitochondrial	2.25	1.60	1.51 × 10^−4^	2.93 × 10^−3^
	AT5G16060	Cytochrome C oxidase biogenesis protein Cmc1-like	2.24	1.48	1.04 × 10^−4^	2.93 × 10^−3^
	AT2G43535	Defensin-like protein 196	1.84	1.58	2.05 × 10^−4^	2.93 × 10^−3^
	AT5G59320	Lipid transfer protein 3	1.85	1.73	3.64 × 10^−3^	1.11 × 10^−2^
	AT3G51600	Lipid transfer protein 5	2.33	2.25	2.40 × 10^−3^	7.03 × 10^−3^
	AT2G02050	NADH-ubiquinone oxidoreductase B18 subunit, putative	2.68	1.89	1.04 × 10^−4^	2.93 × 10^−3^
	AT1G29990	Prefoldin 6	2.35	1.70	1.04 × 10^−4^	2.93 × 10^−3^
	AT5G55125	Ribosomal protein L31	2.18	1.40	1.04 × 10^−4^	2.93 × 10^−3^
	AT2G30410	Tubulin folding cofactor A (KIESEL)	2.25	1.53	1.26 × 10^−4^	2.93 × 10^−3^
	AT5G03660	Unknown Protein	2.29	1.39	1.89 × 10^−4^	6.61 × 10^−3^
	AT3G05070	Unknown protein	2.22	1.52	8.56 × 10^−4^	1.38 × 10^−2^
	AT4G15790	Unknown protein	2.18	1.72	1.40 × 10^−4^	2.93 × 10^−3^
**Decreased Abundance**	AT3G49010	60S ribosomal protein L13-1	−3.14	−2.82	6.80 × 10^−4^	3.29 × 10^−3^
	AT5G63550	DEK domain-containing chromatin associated protein	−1.72	−1.67	4.38 × 10^−4^	2.93 × 10^−3^
	AT2G18020	Embryo defective 2296	−1.99	−1.63	7.50 × 10^−4^	5.76 × 10^−3^
	AT4G30800	Nucleic acid-binding, OB-fold-like protein	−2.11	-	4.55 × 10^−2^	-
	AT3G09500	Ribosomal L29 family protein	−2.28	−1.97	1.76 × 10^−3^	8.87 × 10^−3^
	AT5G23900	Ribosomal protein L13e family protein	−2.48	−2.26	1.53 × 10^−3^	6.24 × 10^−3^
	AT5G64670	Ribosomal protein L18e/L15 superfamily protein	−1.91	−1.72	7.38 × 10^−3^	2.36 × 10^−2^
	AT5G46430	Ribosomal protein L32e	−1.95	−1.79	1.71 × 10^−2^	4.28 × 10^−2^
	AT5G02450	Ribosomal protein L36e family protein	−2.85	−2.41	1.69 × 10^−3^	9.10 × 10^−3^
	AT3G04920	Ribosomal protein S24e family protein	−2.16	−	5.19 × 10^−3^	-
	AT5G20290	Ribosomal protein S8e family protein	−2.35	−1.90	1.79 × 10^−3^	1.25 × 10^−2^
	AT1G52300	Zinc-binding ribosomal protein family protein	−2.48	−2.56	7.14 × 10^−3^	1.35 × 10^−2^

**Table 2:** Top 10 positively co-expressed proteins with the 3 unknown proteins of interest after the drought stress

Unknown Protein	Score	Accession	Annotation
**Protein A**	0.5949	AT5G51940	Non-catalytic subunit of nuclear DNA-dependent RNA polymerases
	0.5940	AT1G11240	Ribosomal RNA-processing protein
	0.5939	AT3G56510	RNA-binding (RRM/RBD/RNP motifs) family protein
	0.5907	AT2G44860 *^§^	Ribosomal protein L24e family protein
	0.5886	AT2G45520	Coiled-coil protein
	0.5810	AT1G79200	Stigma/style cell-cycle inhibitor 1
	0.5739	AT4G27380	Hypothetical protein
	0.5666	AT5G59460	Scarecrow-like transcription factor 11
	0.5617	AT4G37090	UDP-*N*-acetylmuramoyl-l-alanyl-d-glutamate-2,6-diaminopimelate ligase
	0.5563	AT1G16740	Ribosomal protein L20
**Protein B**	0.6690	AT4G30330	Small nuclear ribonucleoprotein family protein
	0.6363	AT2G19720	Ribosomal protein S15A B
	0.6255	AT5G61130	Plasmodesmata callose-binding protein 1
	0.6135	AT4G00810	60S acidic ribosomal protein family
	0.6094	AT3G06680	Ribosomal L29e protein family
	0.6073	AT4G15000 *^§^	Ribosomal L27e protein family
	0.6070	AT3G23390 *	Zinc-binding ribosomal protein family protein
	0.6051	AT4G35950	A member of ROP GTPases gene family-like
	0.6042	AT2G27970	CDK-subunit 2
	0.6030	AT3G59650	Mitochondrial ribosomal protein L51/S25/CI-B8 family protein
**Protein C**	0.5496	AT1G29990*^§^	Prefoldin 6
	0.5419	AT2G18040 *	Peptidylprolyl cis/trans isomerase, NIMA-interacting 1
	0.4990	AT1G29850 *^§^	Double-stranded DNA-binding family protein
	0.4950	AT1G66410	Calmodulin 4
	0.4877	AT5G41210	Glutathione S-transferase theta 1/Glutathione S-transferase tau 12
	0.4871	AT4G35570 *	High mobility group B5
	0.4824	AT3G50360 *^§^	Calmodulin 20/Centrin 2
	0.4790	AT2G29960 *	Cyclophilin 5/peptidylprolyl cis/trans-isomerase 19-4
	0.4570	AT5G46020	28 kDa heat/acid-stable phosphoprotein-like protein
	0.4556	AT4G30480	Tetratricopeptide repeat 1

* Differentially expressed protein in the TMT-labelled group. ^§^ Differentially expressed protein in the TMT-labelled group with at least ±2.0-fold change.

## Supplementary Materials

The following are available online, Table S1: Raw tables of proteins and peptides differentially expressed post-drought treatment. Table S2: Differentially expressed proteins in the TMT-labelled group, Table S3: Differentially expressed proteins in the TMT-labelled group with at least 2-fold change, Table S4: KOBAS analysis of enriched pathways of significant proteins in the TMT-labelled group, Table S5: List of over-represented promoters in the significant proteins of the TMT-labelled group.
